# Comparable outcomes from long and short read random sequencing of total RNA for detection of pathogens in chicken respiratory samples

**DOI:** 10.3389/fvets.2022.1073919

**Published:** 2022-12-01

**Authors:** Salman L. Butt, Henry M. Kariithi, Jeremy D. Volkening, Tonya L. Taylor, Christina Leyson, Mary Pantin-Jackwood, David L. Suarez, James B. Stanton, Claudio L. Afonso

**Affiliations:** ^1^Department of Population Medicine and Diagnostic Sciences, College of Veterinary Medicine, Cornell University, Ithaca, NY, United States; ^2^Exotic and Emerging Avian Viral Diseases Research Unit, Southeast Poultry Research Laboratory, United States National Poultry Research Center, Agricultural Research Service, United States Department of Agriculture, Athens, GA, United States; ^3^Biotechnology Research Institute, Kenyan Agricultural and Livestock Research Organization, Nairobi, Kenya; ^4^BASE_2_BIO, Oshkosh, WI, United States; ^5^Department of Pathology, College of Veterinary Medicine, University of Georgia, Athens, GA, United States

**Keywords:** MinION, MiSeq, Newcastle disease virus, avian influenza virus, infectious bronchitis virus, mycoplasma spp., clinical samples, respiratory disease

## Abstract

Co-infections of avian species with different RNA viruses and pathogenic bacteria are often misdiagnosed or incompletely characterized using targeted diagnostic methods, which could affect the accurate management of clinical disease. A non-targeted sequencing approach with rapid and precise characterization of pathogens should help respiratory disease management by providing a comprehensive view of the causes of disease. Long-read portable sequencers have significant potential advantages over established short-read sequencers due to portability, speed, and lower cost. The applicability of short reads random sequencing for direct detection of pathogens in clinical poultry samples has been previously demonstrated. Here we demonstrate the feasibility of long read random sequencing approaches to identify disease agents in clinical samples. Experimental oropharyngeal swab samples (*n* = 12) from chickens infected with infectious bronchitis virus (IBV), avian influenza virus (AIV) and *Mycoplasma synoviae* (MS) and field-collected clinical oropharyngeal swab samples (*n* = 11) from Kenyan live bird markets previously testing positive for Newcastle disease virus (NDV) were randomly sequenced on the MinION platform and results validated by comparing to real time PCR and short read random sequencing in the Illumina MiSeq platform. In the swabs from experimental infections, each of three agents in every RT-qPCR-positive sample (Ct range 19–34) was detectable within 1 h on the MinION platform, except for AIV one agent in one sample (Ct = 36.21). Nine of 12 IBV-positive samples were assigned genotypes within 1 h, as were five of 11 AIV-positive samples. MinION relative abundances of the test agent (AIV, IBV and MS) were highly correlated with RT-qPCR Ct values (R range−0.82 to−0.98). In field-collected clinical swab samples, NDV (Ct range 12–37) was detected in all eleven samples within 1 h of MinION sequencing, with 10 of 11 samples accurately genotyped within 1 h. All NDV-positive field samples were found to be co-infected with one or more additional respiratory agents. These results demonstrate that MinION sequencing can provide rapid, and sensitive non-targeted detection and genetic characterization of co-existing respiratory pathogens in clinical samples with similar performance to the Illumina MiSeq.

## Introduction

Respiratory diseases are a continual significant threat to the global poultry industry (1). Newcastle disease virus (NDV), infectious bronchitis virus (IBV), avian influenza virus (AIV), *Ornithobacterium rhinotracheale* (ORT) (2), *Mycoplasma synoviae* (MS) and *M. gallisepticum* (MG) have been isolated from different avian species presenting similar clinical respiratory disease (3–7). Co-infections with these microbial pathogens produce respiratory disease complexes and complicate accurate disease diagnosis, when using target-specific approaches (5, 8, 9). For example, a commercial broiler flock, first diagnosed as infected with IBV, based on serological assays, was also co-infected with an atypical velogenic NDV, which was overlooked by relying on a single, target-specific detection approach (8). Experimental studies have shown that vaccine strains of IBV prolonged shedding of low pathogenic AIV (LPAIV) type H9N2 and increased the severity of clinical signs and postmortem lesions (10). Progressive pneumonia is a problem in commercial broiler flocks where ORT and H9N2 were primarily isolated; but it was difficult to establish the primary cause of the disease due to mixed infections (5). Co-infections complicate respiratory disease diagnostics and currently, diagnostic approaches to characterize the co-infecting viral and bacterial respiratory pathogens from chicken samples require both classical and molecular diagnostic tools.

One common approach to identify avian pathogens is isolation in embryonating eggs from specific-pathogen-free (SPF) chickens (11). However, coexistence of avian respiratory pathogens (i.e., NDV and AIV) in the same sample might present a diagnostic problem as it is possible to observe overwhelming growth of one agent over the other during isolation causing a biased characterization of clinical samples (12–14). Furthermore, some microbial pathogens associated with respiratory diseases such as Mycoplasmas are difficult and time consuming to culture under laboratory conditions.

A variety of polymerase chain reaction (PCR)-based rapid multiplexed diagnostic assays have been used for detection and molecular epidemiology of respiratory co-infecting pathogens (15–18). However, these assays were developed for specific pathogens, precluding the possibility of detecting unknown pathogens in the samples (19). Additionally, these conventional assays are sensitive to genetic variation, and mismatches on the pathogen's target sequence can lead to false negative results (20).

For decades, pathogen diagnostics and sequencing have been separate endeavors, with sequencing following diagnostics *via* PCR. Sanger sequencing has historically been the gold standard for sequence-based characterization of pathogens, but this approach is time consuming and expensive for complete identification of coinfecting agents in clinical samples (21, 22). More recently, the next generation sequencing (NGS) platforms have changed this paradigm by providing the possibility for simultaneous diagnostic testing and sequencing of novel and re-emerging pathogens directly from a clinical sample (23). We have recently optimized conditions for efficient detection or multiple respiratory pathogens in poultry by directly sequencing clinical samples with the Illumina short read sequences (24–26). The widespread application of these sequencing platforms for routine diagnostics is still limited due to the associated longer processing time, complex bioinformatics expertise of random sequencing data analysis, and higher cost, hence need for the improving recent alternative diagnostic methods to counter these challenges.

Targeted sequencing on the long-read sequencing platform (Oxford Nanopore Technologies) (27), MinION has recently been used to increase the utility of high-throughput sequencing as a tool for avian pathogen characterization (28, 29). The ability to perform near-real-time sequence analysis of long DNA molecules reduce the time from sample collection to outcome. MinION-based targeted sequencing has been used to genetically type respiratory pathogens such as NDV (30), IBV (31), AIV (32), and infectious laryngotracheitis (ILTV) (33). Recently, a random strand-switching approach was used to identify the novel avian paramyxovirus (APMVs) from cultured samples (34). However, a target-independent, multiplexed, single assay for these respiratory pathogens from uncultured swab samples has not been fully developed. Multiplexed, time- and cost-effective assays that require minimal equipment would be useful in rapidly diagnosing infections and co-infections. In the current study, a multiplexed, random sequencing approach based on MinION nanopore sequencer was developed and compared to RT-qPCR and Illumina MiSeq for the detection of viral and bacterial co-infections in commercial poultry. Additionally, automated bioinformatics pipelines were developed for the rapid characterization of samples by non-experts.

## Materials and methods

### Samples

Clinical oropharyngeal swab samples (hereafter referred to as “clinical samples”) were obtained from chickens from live bird markets in Kenya (*n* = 11) and submitted to the Southeast Poultry Research Laboratory (SEPRL), Athens, Georgia, USA as previously described (35). A second batch of archived chicken oropharyngeal swab samples (*n* = 12) were collected using standard procedures during an experimental coinfection study at SEPRL (hereafter referred to as “experimental samples”) was used in the current study (Supplementary Table 1). Allantois fluid obtained from SPF eggs was used a negative control for both set of samples.

### RT-qPCR assay on experimental swab samples

Total RNA was extracted from 200 μl of each of the virus isolation media used to collect the swab samples using the MagMax RNA extraction kit (Thermo Fisher Scientific, Waltham, MA, USA) as per manufacturer's instructions, and stored at −80°C until further use. The experimental samples were tested by three separate previously described RT-qPCR assays to detect IBV (*spike* gene) (36), AIV (*matrix* gene) (37), and *Mycoplasma* (16S-23S intergenic spacer region) (38) using the AgPath ID, One step RT-PCR kit (Thermo Fisher Scientific, Waltham, MA, USA) performed on the Applied Biosystems 7500 FAST.

### MinION sample preparation

#### cDNA synthesis

For cDNA synthesis, prior to MinION library preparation, a reaction mixture of 11.5 μl of total RNA (~50 ng RNA), 0.5 μl of 250 nM random hexamers (New England Biolabs, Ipswich, MA) and 1 μl of 10 mM dNTPs was incubated at 65 °C for 5 min, chilled on ice for 1 min followed by the addition of 7 μl of cDNA synthesis mix including SuperScript IV (Thermo Fisher Scientific, Waltham, MA, USA) according to the manufacturer's instructions. The reaction mixture was incubated at 23°C for 10 min and, 55°C for 10 min for cDNA synthesis. The reaction was terminated at 80°C for 10 min, and then chilled on ice. To remove residual RNA, the cDNA solution was incubated with 1 μl of RNase H at 37°C for 20 min according to the manufacture's instruction.

#### Second strand DNA synthesis

The cDNA was immediately used for second strand synthesis. Briefly, 20 μl of cDNA solution was mixed with 10 μl of NEBNext (New England Biolabs, Ipswich, MA) second strand synthesis reaction buffer, 5 μl of NEBNext second strand enzyme mix and 45 μl of nuclease free water (NFW), incubated at 16°C for 1 h and cooled at 4°C. dsDNA was purified with AMPure XP beads (Beckman Coulter, Indianapolis, Indiana) at a bead: DNA volumetric ratio of 1.8:1 and eluted in 52 μl of NFW.

#### Adapter ligation

dsDNA was repaired and dA-tailed using 45 μl of dsDNA, 7 μl of Ultra II End-prep reaction buffer, 3 μl Ultra II End-prep enzyme mix (NEBNext Ultra End Repair/dA-Tailing Module, New England Biolabs) and 5 μl Nuclease-free water. The reaction mixture was incubated at 20°C for 5 min and 5 min at 65°C. AMPure bead purification was performed at 1:1 volumetric ratio of beads: DNA according to manufacturer's protocol. 15 μl of end-prepped DNA was mixed with 5 μl barcode adapter (1-96 barcoding kit, ONT) and 20 μl blunt/TA ligase master mix. The reaction mixture was incubated for 15 min at room temperature (RT). The adapter-ligated DNA was purified with AMPure bead at 0.4:1 volumetric ratio of bead: DNA and eluted in 26 μl NFW.

PCR-based barcoding was performed using 25 μl of adapter-ligated dsDNA, 2 μl of barcode and 50 μl of Long-Amp Taq 2X Master mix (New England Biolabs, Ipswich, MA). A reaction mixture of 100 μl was used for amplicon synthesis with the following conditions: denaturation at 95°C for 3 min; 17 cycles of denaturation at 95°C for 15 s, annealing at 62°C for 15 s and extension at 65°C for 90 s, and final extension of 65°C for 90 s and chilled at 4°C.

#### Library preparation and sequencing

The barcoded dsDNA was purified using AMPure beads (bead: DNA, 1:1 volumetric ratio), repaired by dA-tailing end prepped, purified (bead: DNA, 1.6:1), and adapter ligated by using 60 μl of 700 ng pooled (equal volume) barcoded sample, 10 μl of Adapter Mix (AMX 1D), 20 μl of NEBNext Quick Ligation Reaction Buffer (5X) and 10 μl of Quick T4 DNA Ligase (New England Biolabs, Ipswich, MA). The reaction mixture was mixed gently by flicking the tube, and incubated for 10 min at RT. After bead purifying the prepared DNA library was eluted in 15 μl of elution buffer.

A FLO-MIN106 R9.4 flow cell (27) was equilibrated to RT for 10 min and then primed with running buffer as per manufacturer's instructions. The DNA libraries were prepared by combining 12 μL of the library pool with 2.5 μL NFW, 35 μL RBF (Running Buffer Fuel), and 25.5 μL library loading beads. After the MinION Platform QC run, the DNA library was loaded into the MinION flow cell *via* the SpotON port. The standard 1D sequencing protocol was initiated using the MinKNOW software v.5.12.

### MiSeq sample preparation

The same clinical and experimental samples were processed for MiSeq sequencing for a side-by-side comparison of sequencing data obtained from MinION and MiSeq. Briefly, DNA libraries were prepared from total RNA (used for MinION library synthesis) (*n* = 25) using the KAPA Stranded RNA-Seq Kit (Roche Sequencing Solutions, Inc., CA, USA) according to manufacturer's recommendations. Concentrations and distribution sizes (bp) of the cDNA in the KAPA libraries were assessed by Qubit^®^ dsDNA HS Assay Kit (Thermo Fisher Scientific, Waltham, MA), and Agilent 2,100 Bioanalyzer (Agilent technologies Inc., Germany), respectively. Paired-end sequencing of the diluted pooled libraries (10 μl each; 4 nM final concentration) was performed on an Illumina MiSeq platform for 39 h using the 300 cycle MiSeq Reagent Kit v 2 (Illumina, USA) according to manufacturer's instructions (39).

### MinION data analysis

Raw FAST5 data was basecalled, demultiplexed, and trimmed using Guppy 6.1.2, model “dna_r9.4.1_450bps_hac,” barcode set “EXP-PBC096,” with “–detect_mid_strand_barcodes,” and “—trim_barcodes,” without “–require_barcodes_both_ends.” Reads were assigned taxonomic classifications using KrakenUniq (v0.5.8) (40) modified with local patches, against a hierarchical set of databases containing vector/contaminant sequences, host genome (*Gallus gallus* GRCg6a), human genome (GRCh38.p13), and the BASE_2_BIO LLC (Oshkosh, WI, USA) untargeted database of microbial reference sequences. Classifications were further adjusted using a patched version of the “krakenuniq-filter” script, adjusting assignments up the taxonomic tree until the k-mer specificity was 0.05 for viral taxa and 0.25 for all other taxa. Each identified taxon was then further verified by BLASTn (41) search of a random subset of taxon-assigned reads against the full GenBank “nt” database and subsequent lowest common ancestor assignment by in-house tools. For taxa of interest (i.e., NDV, IBV, and AIV), genotypes were called using the standard BASE_2_BIO genotyping module and curated agent databases. *De novo* assemblies of non-host reads were performed using MEGAHIT (v1.2.9) (42) with default settings, minimum contig length of 500 bp. All steps involving read mapping were performed with minimap2 (v2.24-r1122) (43). Tabulation, summarization, and visualization of correlation analysis results was performed in R v4.1.1 (https://www.R-project.org).

### MiSeq data analysis

Analysis of Illumina MiSeq data was performed as described above for the MinION but with the following differences: Data was trimmed using Trim Galore (https://github.com/FelixKrueger/TrimGalore) v06.7 to remove residual adapter sequences and low-quality 3' ends. Steps involving read mapping were performed using BWA MEM (v0.717-r1188) (44) with default settings.

## Results

### Rapid detection of pathogens from clinical samples

A total of twenty-three samples were used in this study, along with two negative controls. Samples 1 to 11 were clinical samples and 13 to 24 were experimental samples (Supplementary Table 1). In Table 1, comparative characterization of the clinical samples with MinION vs. MiSeq are presented as total reads, classified reads (belong to chicken and microbial genomes), the genotype calling based on the coverage breadth (3x) and the identification of major respiratory pathogens which included a selected list of known targeted and non-targeted agents as obtained from the known avian disease literature and identified using >1% of relative read abundance threshold. In addition to the full 8 h MinION run, a subset of reads corresponding to the first hour of the MinION sequencing run were analyzed separately to provide insight into the potential for rapid turnaround times. Prior to sequencing, the clinical samples 1 to 11 were NDV-positive using RT-qPCR targeting the Matrix gene, with Ct values ranging from 12.04 to 36.59. NDV was detected in all 11 samples in the first hour of MinION sequencing. Ten of the 11 samples had sufficient genome sequence coverage to accurately assign the genotype after 1 h–all 11 were correctly genotyped in the MiSeq run. In addition to genotype, determination of the fusion cleavage site amino acid motif and subsequent virulence classification was possible for some, or all samples depending on platform and run time (MiSeq: 11/11; MinION 8 h: 6/11; MinION 1 h: 3/11). The consensus sequences assembled from pathogen-specific reads showed presence of virulent NDV as predicted by the presence of amino acid motif (RRQKR↓F) at the cleavage site of the Fusion (F) protein (Supplementary Table 2). The co-infecting pathogens were present at the arbitrarily designed 1% threshold on at least one of the platforms–however, additional agents were detected in additional samples at levels below this cutoff. All 11 clinical samples contained other known avian respiratory pathogens, which were detected in addition to NDV at significant levels (>1% of total microbial reads), including *Mycoplasma* (*M. gallisepticum, M. synoviae*, and *M. pullorum*), *Avibacterium sp., Gallibacterium sp*., and *Ornithobacterium rhinotracheale*.

**Table 1:** Pathogen identification from MinION and MiSeq sequencing from clinical samples collected from Kenya.

**Sample**	**Total read count**		**Classified read count**		**NDV Ct**	**Called NDV genotype**			**Coverage breadth** ^**a**^			**Major respiratory pathogens^b^**	**Relative abundance (%)** ^**c**^			**Assigned read count**		
	**MiSeq**	**MinION**	**MiSeq**	**MinION**		**MiSeq**	**MinION (1 h)**	**MinION (8 h)**	**MiSeq**	**MinION (1 h)**	**MinION (8 h)**		**MiSeq**	**MinION (1 h)**	**MinION (8 h)**	**MiSeq**	**MinION (1 h)**	**MinION (8 h)**
1	1,735,278	13,090	1,488,350	7,809	13.87	V	V	V	1	0.932	0.997	NDV	27.2	18.8	15.5	383,604	430	982
												Avi	0	1.6	1.6	263	37	100
												MP	1	1.6	1.4	13,723	37	89
												ORT	11.8	15.4	14.6	166,663	352	928
2	1,614,837	14,761	981,135	11,817	15.75	V	V	V	1	0.159	0.619	NDV	3.7	2.3	2.1	34,922	62	141
												Avi	0.2	1.6	1.5	1,497	44	98
												ORT	0.9	2.3	2.6	8,367	64	174
3	1,078,112	38,292	816,517	25,967	17.81	V	V	V	0.945	0	0.024	NDV	0.7	1	0.8	5,776	14	28
												Avi	0.2	9.6	10.6	1,891	137	366
												ORT	0.7	5.2	5.6	5,905	75	195
4	1,500,768	15,871	1,146,857	11,757	16.72	V	V	V	0.735	0.594	0.913	NDV	0.1	3.1	3.2	942	154	375
												Avi	0.9	1.7	1.7	10,426	84	197
												Gal	33.7	31.2	29.8	381,340	1,537	3,449
5	1,860,301	333,690	1,745,836	296,131	12.04	V	V	V	1	0.999	1	NDV	1.9	28.6	29.5	33,521	6,589	20,470
												Avi	1.1	14.1	13.6	18,494	3,243	9,475
6	1,767,122	4,297	1,517,558	3,459	15.43	V	V	V	0.824	0.078	0.316	NDV	0.1	2.4	2.4	1,581	34	78
												Avi	1.2	1.4	1.4	17,998	20	46
7	1,609,048	567,773	835,812	513,686	22.71	V	V	V	0.797	0.159	0.089	NDV	1.1	2.4	1.5	8,861	13	23
												Avi	0.1	9.2	8.2	1,014	50	128
8	1,796,809	299,197	1,484,728	278,030	16.68	V	V	V	1	0.866	0.987	NDV	60.4	34.2	33.5	804,278	361	925
												Avi	0.1	15.3	15.9	812	161	438
9	1,539,612	3,433	1,035,760	1,950	36.59	V	–	–	0.559	0	0	NDV	0.1	0.3	0.1	907	1	1
												Avi	0.8	6	6.8	5,033	19	50
												ORT	0.1	5	4.2	705	16	31
10	1,436,770	75,330	1,094,068	65,267	17.57	V	V	V	1	0.421	0.87	NDV	16.8	19.8	17.1	176,050	134	315
												Avi	0	1.9	1.8	89	13	34
												Gal	0	12.9	12.8	390	87	236
												MG	0.1	1.2	1.5	1,401	8	28
11	1,461,438	6,718	1,117,678	4,962	18.19	V	V	V	0.996	0.891	0.98	NDV	12.1	32.3	34.2	101,597	221	591
												Avi	0.5	1.3	1.2	4,502	9	20
												ORT	0.1	2	2.1	732	14	37
12^d^	N/A	953	N/A	5	N.T.	N/A	–	–	N/A	0	0	NDV	0	50	33.3	N/A	1	1

a As fraction of whole genome at > 3x depth of coverage.

b Known respiratory pathogens detected on either platform at >1% abundance.

c Relative abundance as a fraction of non-metazoan reads.

d No-template control, sequenced only on MinION (no MiSeq data). NT, not tested; NDV, Newcastle disease virus also known as Avian orthoavulavirus; Avi, Avibacterium sp; Gal, Gallibacterium sp; ORT, Ornithobacterium rhinotracheale; MP, Mycoplasma pullorum.

### Rapid detection of pathogens from experimental samples

MinION sequencing of the twelve experimental samples and a negative template control is summarized in Table 2 as total reads, median read length, classified read counts, chicken host reads, fraction of host reads from total reads, read count for each pathogen from 8 to 1 h of sequencing, coverage breadth across the whole microbial genomes (AIV, IBV and MS). The fraction of host reads ranged from 0.80 to 0.96 of the total reads. The non-chicken reads (from 8 to 1 h sequencing run) were classified as microbial reads belonging to IBV, AIV and MS, alone or combined. These experimental samples were also tested using RT-qPCR for the presence of the suspected pathogens, and Ct values for the respective pathogen are included in Table 2. The Ct values for IBV fell in a narrow range from 21.94 to 25.87. IBV reads were detected in all twelve samples in the first hour of MinION sequencing. Coverage read depth was sufficient (3x) to accurately assign IBV genotypes in 9/12 samples after 1 h, and in 12/12 samples after 8 h (as well as in the MiSeq run). For AIV, reads were detected in 10/11 RT-qPCR-positive samples after 1 and 8 h. Full HA/NA subtypes were assigned for 5/11 samples after 1 h, 6/11 after 8 h, and 11/11 in the MiSeq run. For MS, reads were detected after 1 h in 10/10 RT-qPCR-positive samples. No target agents were detected in the mock control. Of all agent/sample combinations, in only one sample contained a single MS-specific read in the full 8 h sequencing run, but the sample was RT-qPCR-negative for the agent.

**Table 2:** Microbial reads obtained at different hours (h) from random sequencing using MinION on experimental oropharyngeal swab samples.

**Sample**	**Total reads**	**Median read length**	**Classified read count**	**Fraction host**	**Read count (8 h)**			**Read count (1 h)**			**Coverage breadth (8 h)** ^**a**^			**Coverage breadth (1 h)**			**RT-qPCR (Ct)**		
					**IBV**	**MS**	**AIV**	**IBV**	**MS**	**AIV**	**IBV**	**MS**	**AIV**	**IBV**	**MS**	**AIV**	**IBV**	**MS**	**AIV**
13	39,951	654	31,385	0.91	137	0	227	34	0	45	0.50	0.00	0.84	0.13	0.00	0.27	24.08	N.D.	22.08
14	45,258	705	39,587	0.92	292	294	2,006	41	49	359	0.84	0.97	0.96	0.12	0.76	0.95	23.15	33.58	18.71
15	22,151	459	14,844	0.63	143	46	17	27	6	2	0.57	0.78	0.12	0.09	0.00	0.00	24.17	33.14	25.63
16	67,481	509	38,080	0.96	159	202	321	31	33	48	0.65	0.94	0.90	0.11	0.62	0.30	24.45	33.91	21.87
17	36,239	488	19,999	0.78	684	3,037	4	114	560	1	0.98	0.99	0.00	0.39	0.98	0.00	22.38	29.34	27.75
18	42,522	576	34,922	0.87	1,360	1,893	3	263	355	2	1.00	0.98	0.00	0.66	0.97	0.00	21.94	30.97	28.79
19	24,190	294	9,007	0.80	262	662	0	56	104	0	0.70	0.98	0.00	0.21	0.92	0.00	22.42	29.87	36.21
20	26,991	470	15,809	0.90	135	1	487	30	0	73	0.62	0.00	0.95	0.04	0.00	0.58	24.02	N.D.	20.92
21	757,670	873	697,572	0.99	328	1,001	237	63	201	36	0.82	0.97	0.90	0.14	0.92	0.21	22.92	29.86	21.84
22	154,062	641	139,353	0.93	100	7,393	0	18	1,356	0	0.40	1.00	0.00	0.01	0.98	0.00	25.87	29.55	N.D.
23	124,015	609	81,368	0.88	954	1,322	727	159	234	123	1.00	0.98	0.95	0.55	0.97	0.75	22.4	31.54	21.48
24	86,752	977	79,936	0.94	736	2,061	4	152	386	1	1.00	0.99	0.00	0.62	0.96	0.00	22.52	30.12	29.17
NTC^b^	53,682	170	21	0.38	0	0	0	0	0	0	0.00	0.00	0.00	0.00	0.00	0.00	N.D.	N.D.	N.D.

a As fraction of whole genome at > 3x depth of coverage.

b No-template control. ND, not detected; IBV, Infectious bronchitis virus; MS, Mycoplasma synoviae; AIV, Avian influenza virus.

### Reference-based and *de novo* genome determination

In addition to detection and genotyping, rapid NGS can provide detailed sequence information on the pathogens found in a sample. For each experimental sample, Table 2 lists the breadth of genome coverage (minimum 3x depth) calculated from read alignment. After 1 h of MinION sequencing, 3/12 samples had IBV coverage breadth > 50%, corresponding approximately with a Ct cutoff of 22. After 8 h, this fraction increased to 11/12. For MS, 9/10 samples had >50% coverage after 1 h, and 10/10 had >75% coverage after 8 h. For AIV, 3/11 samples had >50% coverage after a single hour, while 6/11 had similar coverage after 8 h.

### Correlation of MinION and MiSeq sequencing with RT-qPCR on AIV, IBV, and MS

**Figure 1:**
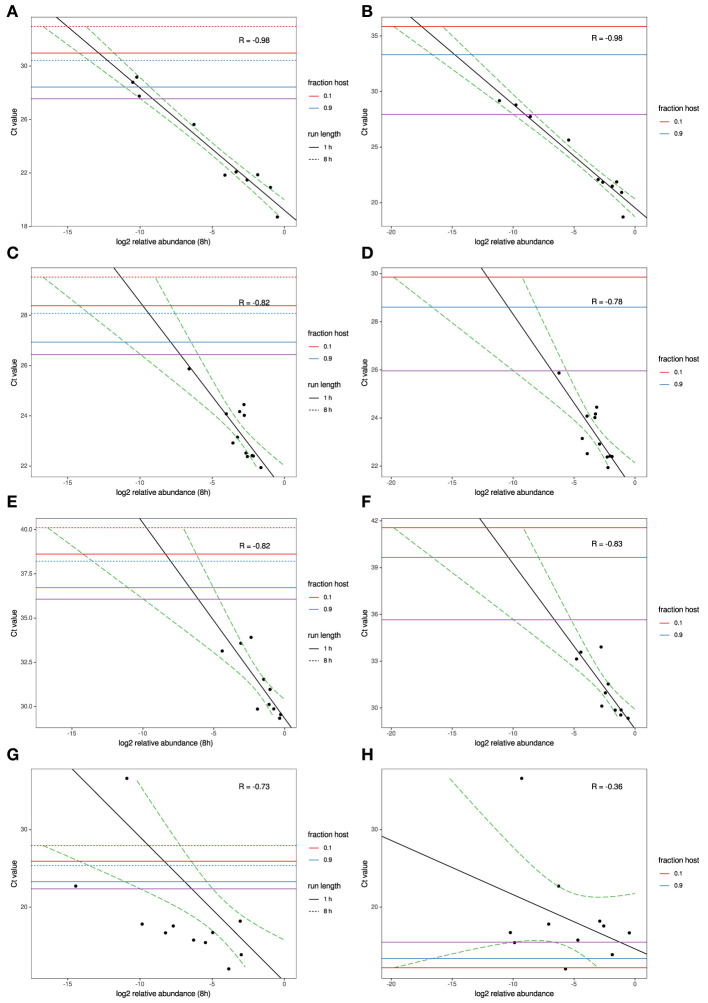
MinION and MiSeq read abundance vs RT-PCR Ct; for Avian Influenza virus **(A,B)**; for Infectious bronchitis virus **(C,D)**; for Mycoplasma synoviae **(E,F)** in experimental samples and for Newcastle disease virus **(G,H)** in clinical samples. Black line indicates best-fit linear regression model. Red/blue horizontal lines mark Ct thresholds at which a single read would be estimated to be observed with 95% confidence at different run times and levels of host contamination, given current experimental conditions, and assuming 12 multiplexed samples. Purple horizontal line marks Ct threshold corresponding to on average one agent read per thousand microbial reads at 95% confidence.

To model the relationship between RT-qPCR Ct values and NGS read abundance, relative read abundance (out of all microbial reads) for each of the four agents with RT-qPCR data available were plotted on a log2 scale against their known Ct values (Figures 1A–H). A linear least-squares model was fit to the data, and the lower 95% confidence interval of this model was used to estimate the lowest Ct value at which an agent read would be detected on average under several different sets of experimental assumptions. This model was also used to estimate the expected Ct value of an agent that is seen at an abundance of one read per thousand microbial reads. The estimated Ct thresholds at which a single read (MinION/MiSeq sequencing) per thousand microbial reads to be observed with 95% confidence at different run times and levels of host contamination, given current experimental conditions, and assuming 12 multiplexed samples was determined to be 27/27.5 for AIV, 26.5/26 for IBV and 36/36.5 for MS (Figures 1A–F, purple horizontal line). The three agents used in the experimental study all have strong correlation between Ct and log2 abundance (between−0.82 and−0.98). IAIV has the strongest correlation, and the largest range of Ct values (19–36) (Figures 1A,B). The other two agents in this study, IBV (Figures 1C,D) and MS (Figures 1E,F), had slightly weaker correlation, but also significant smaller Ct ranges (22–24 and 29–34, respectively).

**Figure 2:**
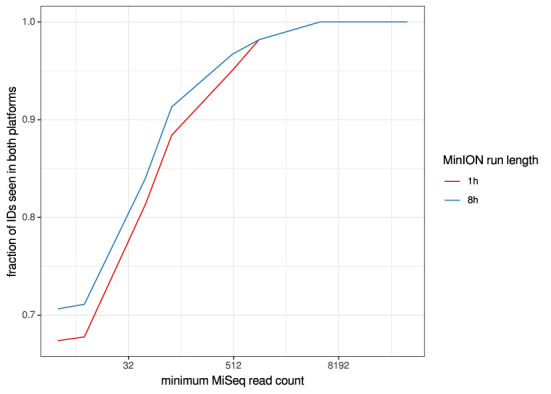
Overlaps of taxon IDs between MiSeq and MinION runs as a function of taxon abundance and MinION run length. A minimum k-mer count of 20 and a minimum b-score (subsampled BLAST agreement) of 0.7 was calculated from IDs.

**Figure 3:**
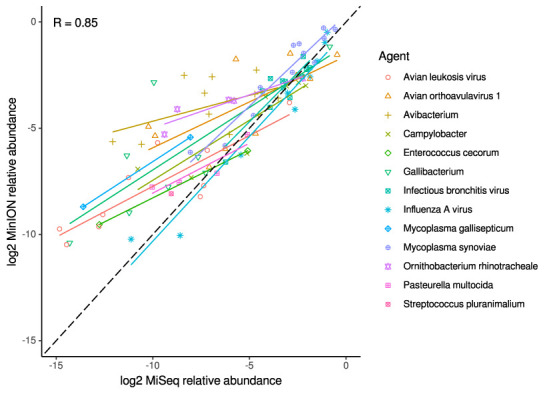
Correlation of per-taxon relative read abundances between Illumina MiSeq and MinION runs. Individual trend lines show least-squares regression models. Dashed black line indicates the identity (1:1) relationship. Data used was from experimental and clinical samples. Pearson's R value is calculated from the combined dataset.

**Figure 4:**
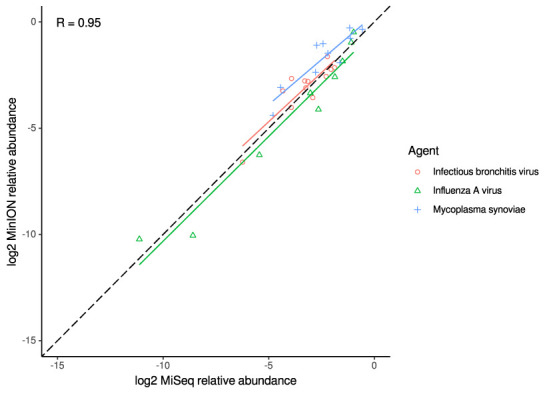
Correlation of per-taxon relative read abundances between Illumina MiSeq and MinION runs of experimental samples. Individual trend lines show least-squares regression models. Dashed black line indicates the identity (1:1) relationship. Pearson's R value is calculated from the combined dataset.

**Figure 5:**
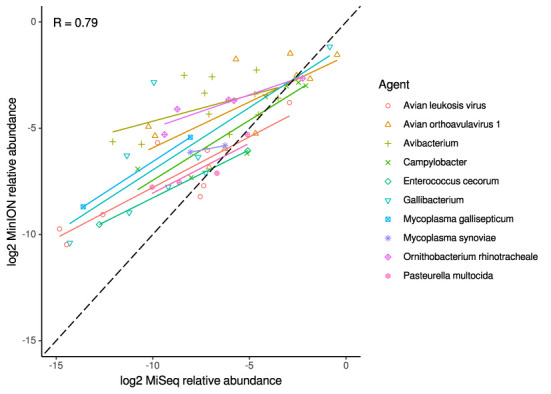
Correlation of per-taxon relative read abundances between Illumina MiSeq and MinION runs of clinical samples. Individual trend lines show least-squares regression models. Dashed black line indicates the identity (1:1) relationship. Pearson's R value is calculated from the combined dataset.

For the clinical samples, correlation of NDV relative abundance and Ct is generally poor (Figures 1G,H). The overlaps of taxon IDs between MiSeq and MinION is demonstrated in Figure 2. Nearly parallel lines representing the number of taxon identification indicated a relationship (as expected) on the overlaps in detection as a function of taxon abundance and MinION run length. The comparison of per-taxon relative read abundances between Illumina MiSeq and MinION sequencing runs in combined clinical and experimental samples showed strong correlation (*R* = 0.85) as the identified taxa are highlighted with different color-coded symbols in Figure 3. Here, for simplicity the comparison was done with a selected number of viral and bacterial respiratory disease-causing agents. In addition, there was extremely high correlation (*R* = 0.95) between relative abundance estimates from MinION and MiSeq sequencing data from the experimental swab samples (Figure 4). The per-taxon relative read abundances comparison between Illumina MiSeq and MinION sequencing runs of clinical samples also showed a moderate correlation (*R* = 0.79) as shown in Figure 5.

## Discussion

Sequence-based pathogen characterization approaches have evolved rapidly and are broadly accepted in the global research community. This study demonstrates the utility of random amplification for untargeted MinION nanopore sequencing to achieve accurate identification and preliminary genetic characterization of viral and bacterial agents in co-infected clinical and experimental samples. We have shown that the MinION platform, as expected produces much smaller read output than MiSeq platform; however, in terms of positive-negative detection and sequence-based agent identification, it can approach the sensitivity of the MiSeq-based approach. This identification of the pathogen, when present at moderate abundance can be achieved with runtimes as short as 60 min, providing advantages in terms of cost, speed of data acquisition and processing time per sample in clinical settings. We have demonstrated strong quantitative correlation both between sequencing platforms and between sequence read abundance and RT-qPCR Ct values, indicating that detection sensitivity of nanopore sequencing is limited primarily by sequencing depth rather than any inherit weakness of the platform. A major advantage of this platform is the ability to adjust run times to suit requirements, even during a run. Therefore, it lends itself to cost optimization by balancing run times, multiplexing depth, and host depletion optimization to achieve target sensitivity levels.

Rapid and accurate detection and characterization of the microbial pathogens present in clinical samples has long been a major goal in diagnostic settings, and numerous advances have been made to improve tests. However, no single diagnostic test is perfect and varying scenarios often require a variety of diagnostic tests. For rapid identification of avian respiratory pathogens, single or multiplexed PCR-based diagnostic assays have been developed and widely used. However, the target-specific nature of these assays makes them vulnerable to failure because of the ability of these pathogens to change, causing false negative results (20). Additionally, PCR-based rapid assays provide limited or no additional genetic information about the detected pathogens. Recently, a strand-switching based random MinION sequencing approach has been used on cultured viral pathogens (45). The work described here is a step further toward rapid characterization, as it demonstrates accurate identification and genetic typing of viral and bacterial pathogens from clinical samples. Also, it is demonstrated that a single targeted assay of the suspected viral pathogen (NDV) would have failed to identify co-infecting pathogens including *A. paragallinarum, M. pullorum*, and *G. anatis*, and which would have remained undetected in these clinical samples without additional testing. However, these bacterial pathogens were detected by untargeted MinION sequencing and in most samples confirmed by MiSeq sequencing.

Although multiplexed PCR based assays have been developed to detect multiple respiratory pathogens in a single assay, this incrementally improved approach still requires a prediction of what pathogens (or genetic variant in case of RNA viruses) are in a sample and will not identify unknown agents, which would result in the incomplete characterization of the clinical samples (8). The presence of more than one pathogen in clinical samples is known to occur and the diversity in the genetic material makes it necessary to perform additional assays to identify some of the pathogens despite the availability of multiplexed assays for respiratory viral pathogens. This MinION sequencing approach is target independent, which may reduce the chances of failure in detection of pathogens due to genetic change. Additionally, the ability to use total RNA (rRNA and mRNA) provides an opportunity to detect pathogens both from genomic viral RNA as well as the rRNAs of replicating bacterial pathogens and mRNA of replicating DNA viruses.

It has been reported that upgraded nanopore sequencing flow cells are capable of achieving as high as 95% raw accuracy (46). It is likely that as the sequencing technology and base calling algorithms will improve the single-read accuracy, further diminishing data analysis challenges specific to noisy long-read data as compared to short-read sequencing. Short-read-based metagenomics studies on platforms such as Illumina have experienced widespread use due to the high accuracy of sequencing. Although Sanger and Illumina (sequencing-by-synthesis) sequencing platforms are considered the gold standard in terms of accuracy (23), these approaches have limitations. Sanger sequencing is necessarily target specific and Illumina-based sequencing shares some similar limitations as MinION in terms of data management. It can become challenging to analyze hundreds of thousands to millions of individual reads due to the computational power and time required. In our current study, an automated pipeline running on cloud resources analyzed each sample in parallel in an average of 2.6 h (minimum 1.5, maximum 4.8) with no user intervention. This workflow overcomes the challenges associated with the lack of computational resources and speed of data analysis.

The primary current limitation of the non-targeted metagenomic sequencing assay is its lower sensitivity compared with targeted amplification. However, this study demonstrates that samples with Ct values into the 30s can be reliably detected from randomly amplified samples in as little as 1 h of sequencing time, often with depth sufficient to yield the genotypic classification. This observation is supported by linear modeling of NGS abundance and RT-qPCR thresholds, which backs the conclusion that at least some agents with Ct values > 30 should be reliably identifiable under similar experimental conditions. The sensitivity of detection in these experimental samples was hampered both by a high degree of host contamination, which varied from 63 to 99% in the experimental MinION run, as well as multiplexed library sizes that varied by several orders of magnitude. Improvements in handling these challenges would be expected to significantly increase the sensitivity beyond that already observed and improve the reliability of the approach as a diagnostic tool.

One of the biggest challenges in sequencing-based diagnostics is the presence of nucleic acids in clinical samples from both host and pathogens, which may be sourced from a variety of genomic material. The total RNA sequencing approach adopted in this study allows the capture of broad population of RNAs from the clinical oral swab samples. Although oropharyngeal swab samples have comparatively lower host and commensal bacterial populations, there is still the background of many chicken-reads. Because clinical samples were collected at different time points and the quality of RNA may be low as well, these samples should be sequenced for around 8 h. It is notable that although no pre-enrichment approach was used to detect microbial RNA in the clinical samples, only 60 min of MinION sequence data was sufficient to detect all the test co-infecting viral and bacterial pathogens from the experimental samples. Specific reduction in the rRNA of chicken host will further increase the utility of this approach in recovery of viral genomes from metagenomic samples. At the moment, several companies offer kits for elimination of host and bacterial ribosomal RNAs, and the utilization of those are likely to improve the sensitivity of detection of pathogens (47). An RNaseH approach with probes targeted to rRNA from both chickens and bacteria has shown a significant increase in sensitivity and can be potentially used with this MinION approach as well (24). The utility of the assay for other species has not been tested but this protocol does not include any pre-enrichment of pathogen RNA which could also make it very useful for human pathogens as well.

## Conclusions

The presence of important viral and bacterial pathogens in respiratory clinical samples of chickens was detected by direct extraction of total RNA followed by the use of Oxford Nanopore MinION or Illumina MiSeq sequencing technologies. NDV and various bacterial respiratory agents were detected in chickens from Kenyan live bird markets with both technologies. The MinION platform provided a rapid but still accurate characterization of the co-infecting viral and bacterial pathogens in experimental swab samples. Extensive testing on diverse clinical samples will further evaluate the viability of this protocol for diagnostic settings. In addition, because this MinION-based approach provides for rapid, multiplexed, and cost-effective detection of viral and bacterial pathogens in clinical samples with sufficient sensitivity for many applications, it represents a legitimate alternative for diagnostic laboratories that cannot afford more expensive equipment for next-generation diagnostics. Based on this work and related studies, the goal of a cost-effective, sensitive, and untargeted NGS-based diagnostic tool appears one step closer to reality.

## Data availability statement

These dataset in this study are deposited in the SRA repository under BioProject PRJNA900571 and the accession numbers: SRR22262942- SRR22262990. These datasets can be accessed at link below: https://www.ncbi.nlm.nih.gov/sra/PRJNA900571.

## Author contributions

SB processed the egg-grown and clinical samples, created the MinION libraries, analyzed the MinION data, and wrote the manuscript. HK contributed to the preparation and analysis of NGS data and manuscript preparation. JV developed the MinION data analysis workflow and assisted with manuscript preparation. TT and CL helped with the RT-qPCR for IBV, AIV, and MS. MP-J, DS, and JS assisted in data interpretation and manuscript preparation. CA was involved in the design of the study, data analysis, data interpretation, and writing of the manuscript. All authors were involved with editing the manuscript, read, and approved the final manuscript.

## Funding

This project was supported by USDA CRIS 6040–32000-072.

## Conflict of interest

The authors declare that the research was conducted in the absence of any commercial or financial relationships that could be construed as a potential conflict of interest.

## Publisher's note

All claims expressed in this article are solely those of the authors and do not necessarily represent those of their affiliated organizations, or those of the publisher, the editors and the reviewers. Any product that may be evaluated in this article, or claim that may be made by its manufacturer, is not guaranteed or endorsed by the publisher.

## Supplementary material

Supplementary Table 1 Details of clinical and experimental oropharyngeal swab samples.

Supplementary Table 2 Details of comparison of *de novo* contigs assigned per taxon from MinION and MiSeq sequencing platforms.
