# Supplementary material for: Comparable outcomes from long and short read random sequencing of total RNA for detection of pathogens in chicken respiratory samples
Source: Front Vet Sci. 2022 Dec 1;9:1073919. doi: 10.3389/fvets.2022.1073919 (PMC9751482; doi:10.3389/fvets.2022.1073919)
Supplement: Supplementary Table 1 — Details of clinical and experimental oropharyngeal swab samples. [file Table_1.DOCX]

**Supplementary table 1.** Details of clinical and experimental oropharyngeal swab samples

| **Sample #** | **Sample description** | **Bird type** | **Country/**  **Location** | **Type of sample** | **Clinical/**  **Experimental** |
| --- | --- | --- | --- | --- | --- |
| 1 | 0303-3-M Bird # 1 OP | Male back yard chicken | Kenya | Oropharyngeal | Clinical |
| 2 | 0303-3-M Bird # 7 OP | Male back yard chicken | Kenya | Oropharyngeal | Clinical |
| 3 | 0303-3-M Bird # 16 OP | Male back yard chicken | Kenya | Oropharyngeal | Clinical |
| 4 | 0303-3-M Bird # 22 OP | Male back yard chicken | Kenya | Oropharyngeal | Clinical |
| 5 | 0303-3-M Bird # 37 OP | Male back yard chicken | Kenya | Oropharyngeal | Clinical |
| 6 | A4/97/O | Male back yard chicken | Kenya | Oropharyngeal | Clinical |
| 7 | 303-1-F Bird # 3 OP | Female back yard chicken | Kenya | Oropharyngeal | Clinical |
| 8 | 303-1-F Bird # 4 OP | Female back yard chicken | Kenya | Oropharyngeal | Clinical |
| 9 | 303-1-F Bird # 66 OP | Female back yard chicken | Kenya | Oropharyngeal | Clinical |
| 10 | 303-1-M Bird # 34 OP | Male back yard chicken | Kenya | Oropharyngeal | Clinical |
| 11 | 303-1-M Bird # 37 OP | Male back yard chicken | Kenya | Oropharyngeal | Clinical |
| 12 | Negative control | Specific pathogen free broilers | USDA-SEPRL | Allantois fluid | Experimental |
| 13 | AIV, IBV | Specific pathogen free broilers | USDA-SEPRL | Oropharyngeal | Experimental |
| 14 | AIV, IBV, MS | Specific pathogen free broilers | USDA-SEPRL | Oropharyngeal | Experimental |
| 15 | AIV, IBV, MS | Specific pathogen free broilers | USDA-SEPRL | Oropharyngeal | Experimental |
| 16 | AIV, IBV, MS | Specific pathogen free broilers | USDA-SEPRL | Oropharyngeal | Experimental |
| 17 | AIV, IBV, MS | Specific pathogen free broilers | USDA-SEPRL | Oropharyngeal | Experimental |
| 18 | AIV, IBV, MS | Specific pathogen free broilers | USDA-SEPRL | Oropharyngeal | Experimental |
| 19 | AIV, IBV, MS | Specific pathogen free broilers | USDA-SEPRL | Oropharyngeal | Experimental |
| 20 | AIV, IBV | Specific pathogen free broilers | USDA-SEPRL | Oropharyngeal | Experimental |
| 21 | AIV, IBV, MS | Specific pathogen free broilers | USDA-SEPRL | Oropharyngeal | Experimental |
| 22 | IBV, MS | Specific pathogen free broilers | USDA-SEPRL | Oropharyngeal | Experimental |
| 23 | AIV, IBV, MS | Specific pathogen free broilers | USDA-SEPRL | Oropharyngeal | Experimental |
| 24 | AIV, IBV, MS | Specific pathogen free broilers | USDA-SEPRL | Oropharyngeal | Experimental |
| 25 | Negative control | Specific pathogen free broilers | USDA-SEPRL | Allantois fluid | Experimental |

**Abbreviations:** AIV; avian influenza virus, IBV; infectious bronchitis virus, MS; Mycoplasma synoviae.
